# Genetic Manipulation of MicroRNAs in the Silk Gland of Silkworm, *Bombyx Mori*

**DOI:** 10.1186/s12575-019-0102-4

**Published:** 2019-08-15

**Authors:** Wei Wang, Xinran Wang, Xuemei Li, Qian Pu, Chengyi Luo, Lili Xu, Xinyue Peng, Shiping Liu

**Affiliations:** 1 State Key Laboratory of Silkworm Genome Biology,Biological Science Research Center, Southwest University, Chongqing, 400715 People’s Republic of China; 2 Chongqing Key Laboratory of Sericultural Science, Chongqing Engineering and Technology Research Center for Novel Silk Materials, Southwest University, Chongqing, 400715 People’s Republic of China

**Keywords:** *Bombyx mori*, Silk gland, microRNA, Functional study, Knockout, CRISPR/Cas9

## Abstract

**Background:**

MicroRNAs (miRNAs) are a class of non-coding RNAs with important post-transcriptional regulatory functions. To reveal the function of miRNAs *in vivo*, the critical step is to change their expression levels in the tissues or organs. In this work, we explored the application of several important genetic techniques in altering the expression of silk gland-specific miR-274 of silkworm (*Bombyx mori*).

**Results:**

Injection of synthesized microRNA mimics and antagomirs exerted no effect on the expression of miR-274 in the silk gland, miR-274 sponge specifically absorbed miR-274 and down-regulated its expression, transgenic overexpression of miR-274 precursor significantly up-regulated miR-274, and finally tissue-specific CRISPR/Cas9 system achieved deletion of miR-274.

**Conclusions:**

A practical technical system was established for studying the functions of miRNAs in silk gland of *Bombyx mori*. Our research provides methodological support for the functional study of miRNAs and other noncoding RNAs in the silk gland and more organs in other species.

## Background

Silkworm is not only an important economic insect, but also a model insect for molecular biology research [1]. Silk gland of silkworm can be divided into three divisions according to its morphology and function [2]. Anterior silk gland (ASG) is the site where liquid silk protein is assembled into silk fibers, middle silk gland (MSG) consists of three segments and synthesizes different sericin proteins, posterior silk gland (PSG) is a coiled long tube and synthesizes silk fibroin proteins. The division-specific structures and functions are highly related to the expression patterns of genes [2, 3]. Therefore, silk gland is an ideal organ for studying gene regulation and tissue remodeling [2].

MicroRNA (MiRNA) is a kind of non-coding RNA post-transcriptionally regulating almost all important life processes [4, 5]. As early as 2010, we identified miRNAs in the MSG and PSG of silkworm at day 3 of the fifth instar (D3 IL5) [6, 7], and confirmed that miR-274 is silk gland-specifically expressed [7]. In 2018, Qin et al. identified the miRNAs related to silk protein synthesis by sequencing the silk glands of three silk-producing strains [8]. So far, however, studies on silk gland miRNAs still mainly focus on the validation of miRNAs and their targets [9, 10], and very few reports are available on their roles in the silk gland, which is mainly due to the lack of effective techniques compatible with this specialized organ.

The techniques for the functional study of miRNAs can be divided into two main categories. One is the strategy of gain of function using miRNA mimics, recombinant virus overexpression vector and transgenic overexpression vector. MiRNA mimic is a chemically synthesized double-stranded RNA to up-regulate miRNAs at cellular and individual levels [11, 12]. Both recombinant viral and transgenic methods are adopted to overexpress the miRNA precursor, which is then successively cleaved by host nucleases to form mature sequences [13, 14]. The second is the strategy of loss of function by means of miRNA antagomir, miRNA sponge and miRNA knockout. MiRNA antagomir is a chemically synthesized sequence reverse complementary to the miRNA and chemical modifications are needed to enhance the stability and transmission efficiency [15]. MiRNA sponge contains tandem repetitive sequences reverse complementary to the miRNA and can reduce the chance of Ago protein in cleaving the target mRNA [16]. CRISPR/Cas9 is an evolved defense mechanism acquired by bacteria and archaea to resist the invasion of viruses or exogenous DNA [17, 18]. Endogenous or artificial gRNA guides Cas9 endonuclease to cleave the double strand DNA at a fixed point in the genome, and in the process of non-homologous end-repair, code-shifting mutations probably happen in coding genes [19]. However, knockout of miRNAs cannot be realized through code-shifting mutation [20–22]. Taking the silk gland-specific miR-274 as an example, here we aim to explore the genetic tools that can effectively manipulate miRNAs in the silk gland, and hopefully provide technical support for the functional study of miRNAs in silk glands.

## Results

### MiR-274 is Exclusively Highly Expressed in the Silk Gland of Silk Worm

**Fig. 1:**
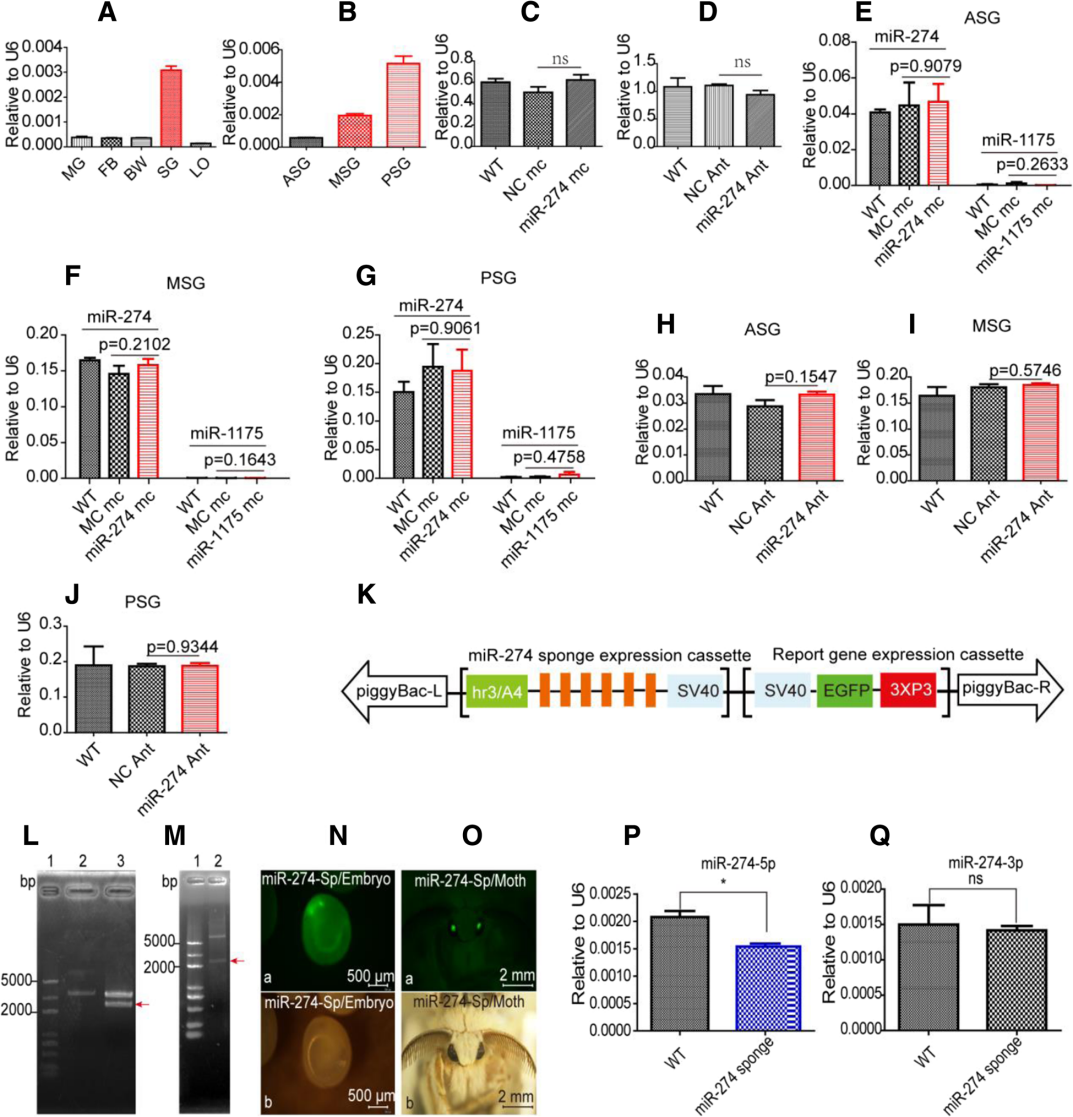
Expression of silk gland-specific miR-274 couldn’t be changed by functional reagents but was down-regulated by miRNA sponge. **a** Spatial expression of miR-274 at D3 IL5. **b** Expression in the three divisions of silk gland. **c** Expression post injection of miR-274 mimics. **d** Expression post injection of miR-274 antagomir. **e**-**g** Expression of miR-274 in different divisions of the silk gland post injection of miR-274 mimic and miR-1175 mimic. **h**-**j** Expression of miR-274 in different divisions of the silk gland post injection of miR-274 antagomir and miR-1175 antagomir. **k** Schematic diagram of transgenic miR-274 sponge vector. **l** Verification of *pSL1180*[*Hr3/A4-miR-274 sponge*] by *Asc* I. 1: Marker; 2: Recovered vector backbone of *pSL1180*[*Hr3/A4*]; 3: Digestion product of *pSL1180[Hr3/A4-miR-274-sponge*]. (**m**) Verification of *piggyBac[3 × P3-EGFP, Hr3/A4–274-sponge]* by *Asc* I. **n** Screening of positive transgenic miR-274 sponge at embyo stage. (a) Observed under blue light; (b) Observed under white light. **o** Screening of positive transgenic miR-274 sponge at adult stage. (a) Observed under blue light; (b) Observed under white light. **p** Expression of miR-274 in the transgenic miR-274 sponge. **q** Expression of miR-274-3p in the transgenic miR-274 sponge. Data represent three biological replicates with three technical replicates and are shown as mean ± SEM. ns, not significant, *P < 0.05

We examined the expression of miR-274 in different tissues of silkworm at D3 IL5 and found that it was not or very lowly expressed in the midgut, fat body, body wall and the left over but highly expressed in the silk gland (Fig. 1a), consistent with our previous results [6]. Its expression level obviously differed in the three divisions of silk gland, most highly expressed in the PSG, and secondly in the MSG (Fig. 1b). The unique expression pattern suggests that this miRNA might play an important regulatory role in the silk gland.

### MiRNA Mimics and Antagomir Failed in Changing the Expression of MiR-274 in the Silk Gland

The silk gland was collected at 48 h post injection of functional reagents for qPCR assay, but both miR-274 mimic and miR-274 antagomir failed in changing the expression of miR-274 in the silk gland (Fig. 1c and d). We tried different concentrations and doses, but still observed no significant changes of miR-274 expression. Further, both miR-274 mimic and antagomir exerted no clear effect in the three divisions of silk glands (Fig. 1e-j). We also injected the functional reagents of some other miRNAs but did not found significant changes in their expressions in the silk gland. For example, the mimic of aae-miR-1175, which significantly changed the expression of miR-1175 in the midgut of mosquitoes [23], also failed in the silk gland of silkworm (Fig. 1e-g). These exogenous molecules might not enter the highly specialized silk gland, or, there possibly exist a special mechanism in the silk gland cells reducing the efficacy of these reagents. Therefore, it is not feasible to study the functions of miRNAs in silk glands by injecting exogenous miRNA mimics and antagomirs.

### Down-regulation of MiR-274 in Silk Gland by Transgenic MiRNA Sponge

The designed miR-274 sponge sequence (Fig. 1k) was cloned into *pSL1180* [*Hr3/A4-Luc*] vector to form the expression cassette of [*Hr3/A4–274-sponge*] (Fig. 1l). The expression cassette was digested with *Asc* I, and cloned into the vector backbone *piggyBac* [*3 × P3-EGFP*] to form the transgenic plasmid *piggyBac* [*3* × *P3-EGFP, Hr3/A4–274-sponge*] (Fig. 1m). The positive F1 individuals were screened under fluorescence microscopy by identifying the green light in the eyes of day 6 embryos (Fig. 1n). The positive F2 generation was obtained from the oviposition of F1 (Fig. 1o). The silk glands of F2 were collected at D3 IL5 for RNA extraction and qPCR assay. The results showed that the expression of miR-274 was down-regulated by about 20% in the [*miR-274-Sponge*] strain (Fig. 1p), and as a control, the expression of miR-274-3p remained unchanged (Fig. 1q). Therefore, miRNA sponge adsorption technique can be explored to down-regulate miR-274 in silk glands, whereas the extent of down-regulation needs to be improved by using more effective promoters.

### Transgenic Overexpression of MiR-274 in Different Divisions of Silk Gland

**Fig. 2:**
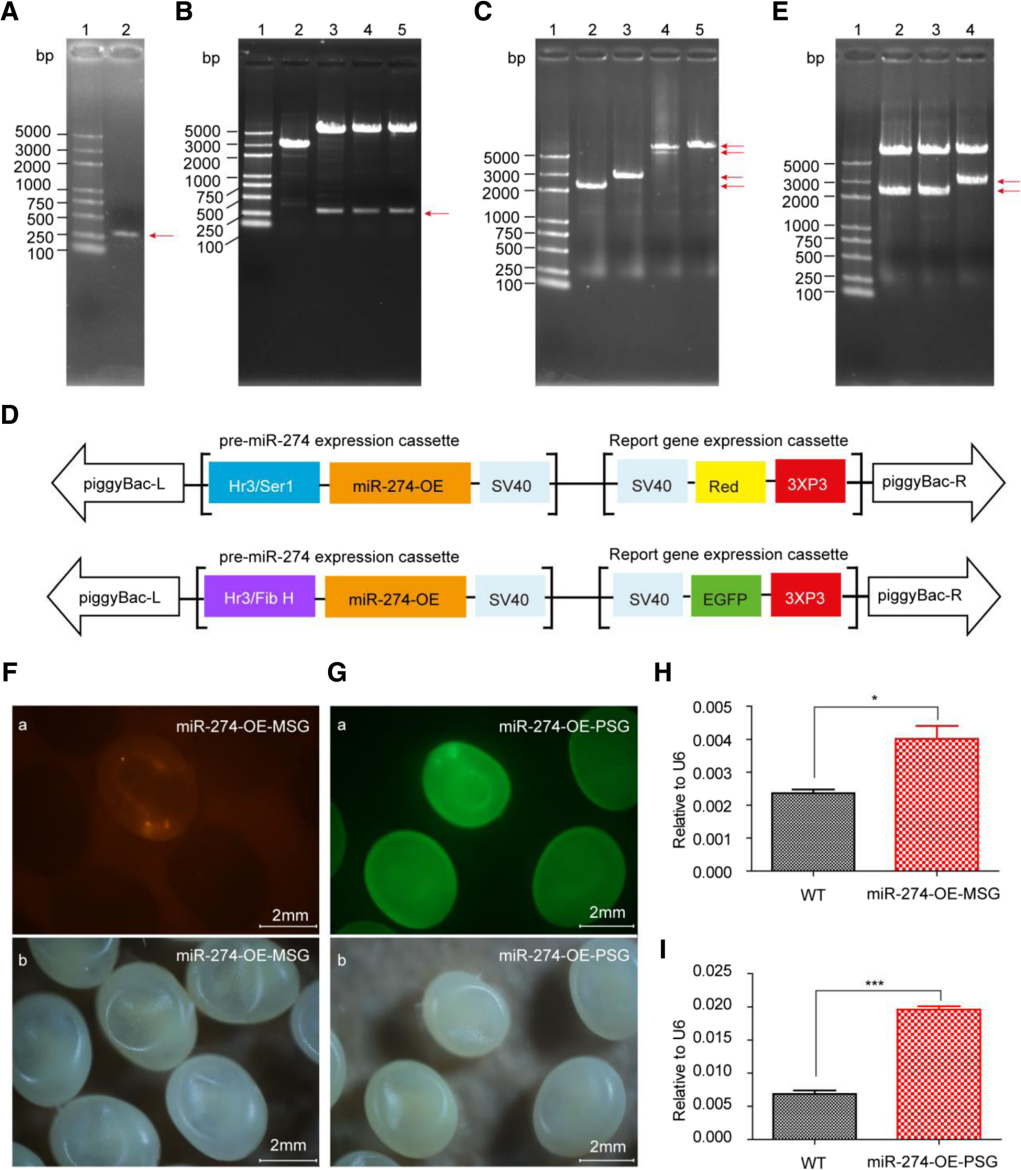
Transgenic overexpression of miR-274 in the silkworm silk gland. **a** PCR product of the sequence containing miR-274. 1: Marker; 2: PCR product. **b** Verified plasmids *pSL1180*[*Hr3/Ser1-miR-274*] and *pSL1180*[*Hr3/FibH-miR-274*] by *BamH* I/*Not* I. 1: Marker; 2. Verification of *pSL1180*[*Hr3/Ser1-miR-274*]; 3–5. Verification of *pSL1180*[*Hr3/FibH-miR-274*]. **c** Expression cassette of miR-274 and the vector backbone *piggyBac* recovering.1: Marker; 2: Gel-recovered [*Hr3/Ser1-miR-274*]; .3: Gel-recovered [*Hr3/FibH-miR-274*]; 4: Gel-recovered vector backbone *piggyBac*[*3 × P3-Red*]; 5: Gel-recovered vector bankbone *piggyBac*[*3 × P3-EGFP*]. **d** Shecmatic diagram of MSG-specific transgenic overexpression vector of miR-274 and PSG-specific transgenic overexpression vector of miR-274. **e** Transgenic overexpression plasmid was verified by single enzyme digestion. 1: Marker; 2: Digested product of *piggyBac*[*3 × P3-Red*] with *Asc* I; 3: Digested product of [*Hr3/Ser1-miR-274-SV40*] with *Asc* I; 4: Digested product of *piggyBac*[*3 × P3-Red, Hr3/FibH-miR-274-SV40*] with *Asc* I. **f** Screening of positive individuals of miR-274-OE-MSG. (a) Observed under green light; (b) Observed under white light. **g** Screening of positive individuals of miR-274-OE-PSG. (a) Observed under blue light; (b) Observed under white light. **h** Expression of miR-274 in miR-274-OE-MSG. **i** Expression of miR-274 in miR-274-OE-PSG. Data represent three biological replicates with three technical replicates and are shown as mean ± SEM. *P < 0.05; ***P < 0.001

The sequence with a total of 279 bp, including 83 bp upstream of pre-miR-274, and 101 bp downstream of pre-miR-274, was PCR-amplified from the miR-274 locus of genome (Fig. 2a), and named miR-274-OE, which was then ligated to the vector backbones *pSL1180*[*Hr3/Ser1*] and *pSL1180* [*Hr3/FibH*] to generate recombinant plasmids *pSL1180*[*Hr3/Ser1-miR-274-OE*] and *pSL1180*[*Hr3/FibH-miR-274-OE*], respectively (Fig. 2b). After double-enzyme digestion and sequencing verification, the recovered expression cassettes [*Hr3/Ser1-miR-274-OE*] and [*Hr3/FibH-miR-274-OE*] were cloned into the transgenic vectors *piggyBac* [*3 × P3-Red*] and *piggyBac*[*3* × *P3-EGFP*], respectively (Fig. 2c), forming the MSG-specific recombinant overexpression plasmid *piggyBac*[*3 × P3-Red, Hr3/Ser1-miR-274-OE*] and the PSG-specific recombinant overexpression plasmid *piggyBac*[*3 × P3-EGFP, Hr3/FibH-miR-274-OE*] (Fig. 2d), and both were finally confirmed by double-enzyme digestion (Fig. 2e). The positive F1 individuals were screened at day 6 embyro in that eyes with red light were miR-274-OE-MSG, and eyes with green light were miR-274-OE-PSG (Fig. 2f and g). The silk glands were collected at D3 IL5 for qPCR assay. The results showed that miR-274 was significantly overexpressed in both MSG and PSG, and detailedly, up-regulated by 70% in miR-274-OE-MSG (Fig. 2h) and by 186% in miR-274-OE-PSG (Fig. 2i). Together, it is feasible to up-regulate miRNAs in the silk gland when the proper promoter is adopted in the transgenic overexpression technology.

### Knockout of MiR-274 in PSG Using RNA-guided CRISPR/Cas9 System

**Fig. 3:**
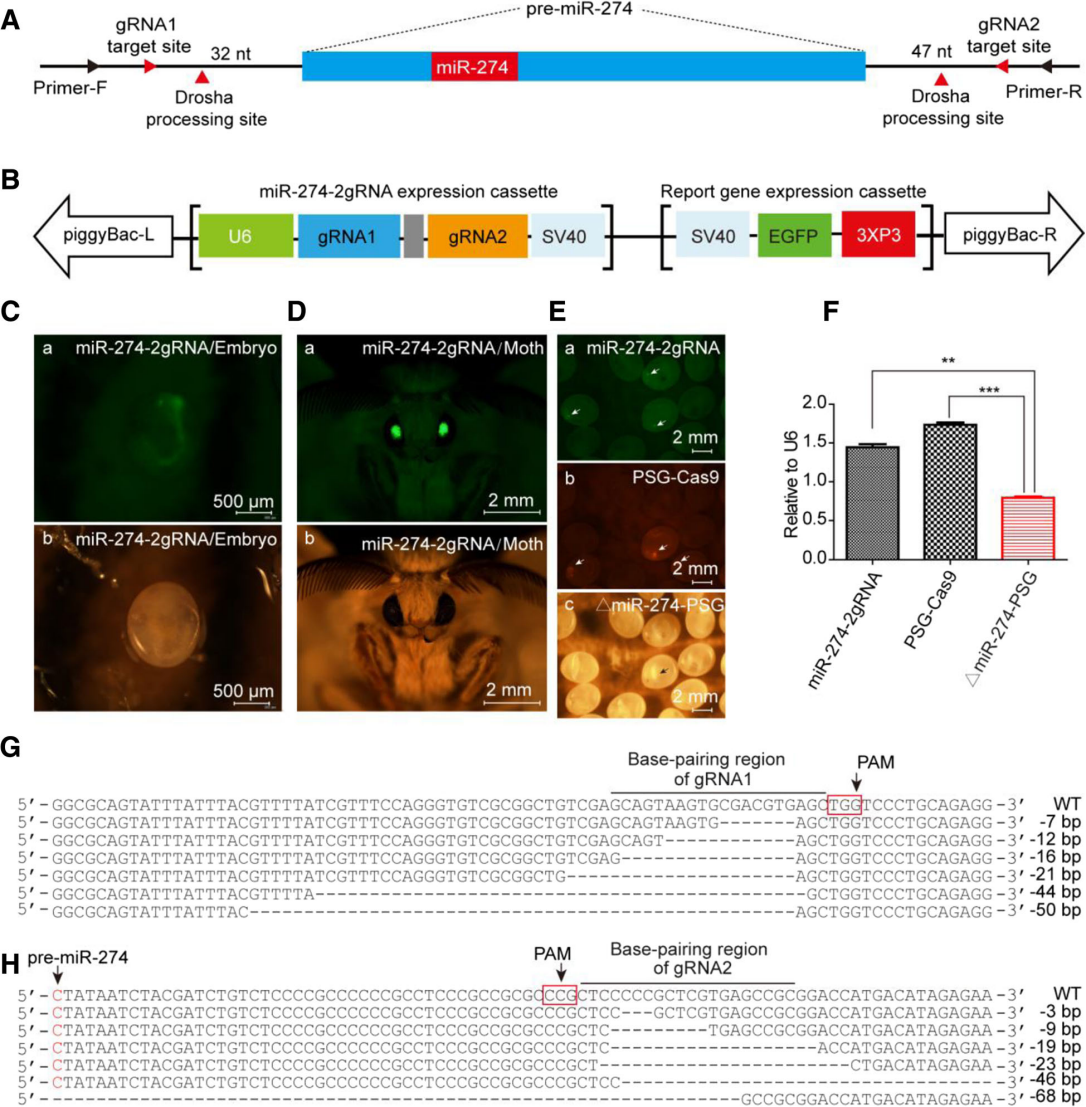
RNA-guided CRISPR/Cas9 to knock out miR-274 in PSG. **a** Design of gRNA. The gRNA site and Drosha site are shown in red arrows. **b** Schematic diagram of transgenic 2gRNA overexpression vector. **c** Screening of positive miR-274-2gRNA at embryo stage. (a) Observed under blue light; (b) Observed under white light. **d** Screening of positive miR-274-2gRNA at adult stage. (a) Observed under blue light; (b) Observed under white light. **e** Screening of positive miR-274 knockout individuals. (a) Screening of positive miR-274-2gRNA under blue light at embryo stage; (b) Screening of positive Cas9 under green light at embryo stage. (c) Positive miR-274 knockout individuals. **f** Expression of miR-274 in the PSG of knockout strain. **g** Base deletion at the site of gRNA1. **h** Base deletion at the site of gRNA2

Our laboratory colleagues have created a transgenic silkworm which specifically expresses Cas9 in the PSG using the *Fib H* promoter, and have successfully deleted the *BmLMN* gene in the PSG through crossing with the transgenic strain expressing gRNA [24]. This PSG-specific CRISPR/Cas9 system is useful for functional study of lethal genes in the silk gland. However, miRNAs cannot be knocked out by code-shifting mutations. Therefore, we determined the gRNA near the Drosha processing site at each end of the precursor of miR-274 (Fig. 3a), hoping that the two gRNAs could be expressed simultaneously to delete the fragments between them.

The synthesized gRNA1 and gRNA2 spacer sequences were sequentially annealed and ligated to *pUC57*[*U6-2gRNA*] to form *pUC57*[*U6-miR-274-2gRNA*] vector. The expression cassette [*U6-miR-274-2gRNA*] was cloned into the transgenic vector *piggyBac*[*3* × *P3-EGFP*], generating the transgenic 2gRNA expression plasmid, *piggyBac*[*3 × P3-EGFP,U6-miR-274-2gRNA*] (Fig. 3b), which was then injected into the eggs for hatching. The positive 2gRNA-expressing transgenic individuals (miR-274-2gRNA) were screened at embryonic and adult stages according to the green light in the eyes (Fig. 3c and d). The adults of miR-274-2gRNA were crossed with those of PSG-specific Cas9 to obtain F1 generation, which had four different light-emitting forms in the eyes (Fig. 3e). The larvae of each group were cultivated to D3 IL5, when the PSGs were collected to extract the total RNA and genome DNA. The q-PCR results showed that miR-274 was successfully knocked out by CRISPR/Cas9 system, and its expression decreased by about 54% in the PSG (Fig. 3f). Through TA cloning and sequencing, it was found that CRISPR/Cas9 system mainly mediated base deletion at target sites of gRNAs, ranging from 7 bp to 50 bp at gRNA1 (Fig. 3g) and from 3 bp to 68bp at gRNA2 (Fig. 3h), respectively. In all examined sequencing results, we noticed some base deletions by two gRNAs, but the fragment deletions between the two gRNAs were not observed.

## Discussion

**Fig. 4:**
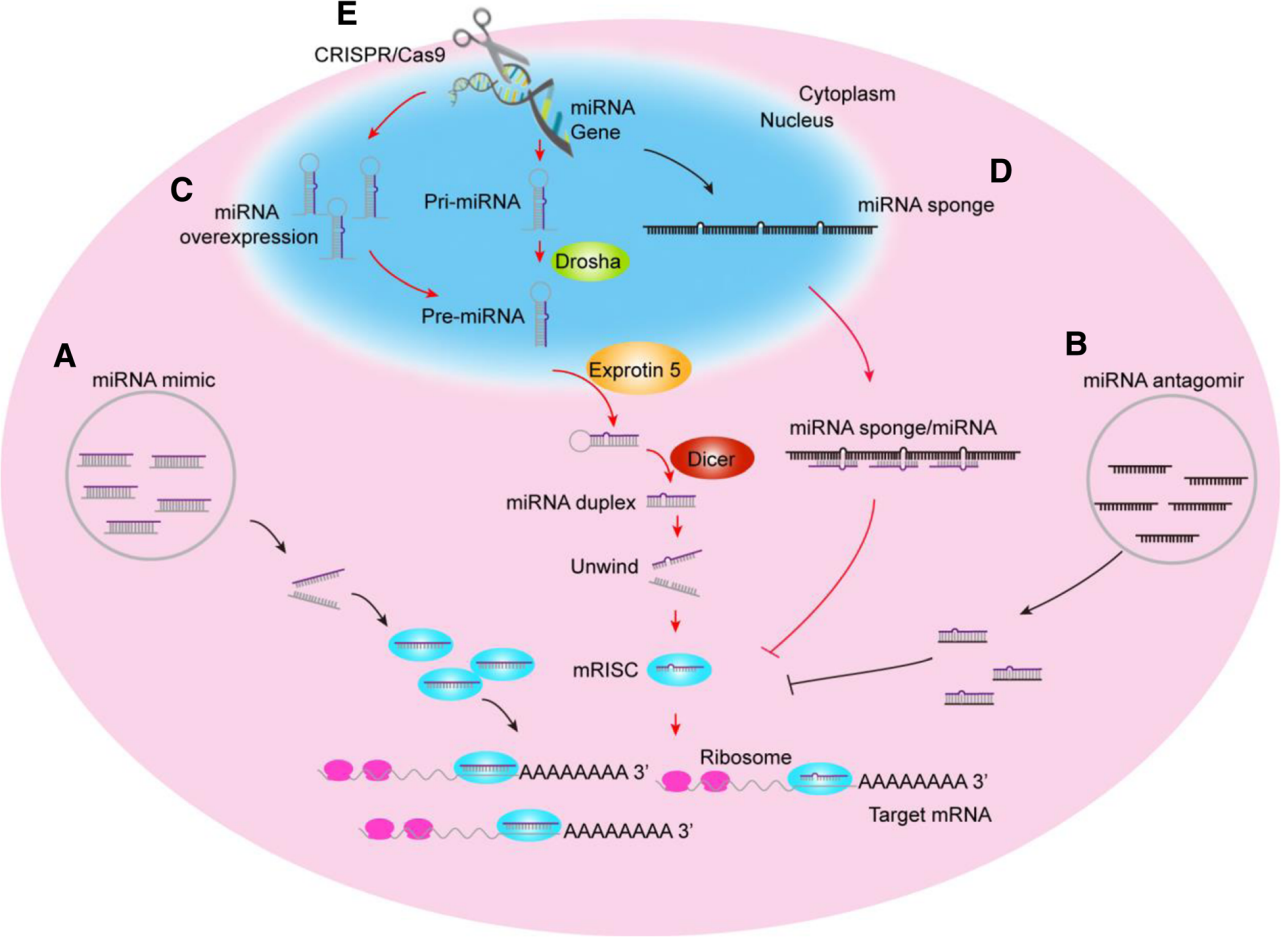
Summary of techniques to change the expression of miRNAs. **a** Up-regulation by miRNA mimic. **b** Down-regulation by miRNA antagomir. **c** Overexpression via transgenic technique. **d** Down-regulation using miRNA sponge. **e** Knockout through CRISPR/Cas9 system

The miRNAs in silk glands, especially those highly expressed in this special organ, are likely to be involved in the regulation of silk gland development and silk protein synthesis. However, how to effectively change their expressions in silk glands is still a challenge. Therefore, we explored the application of five methods to alter the expression of miRNAs in the silk gland (Fig. 4). In our previous work, we revealed the biological significance of miR-1174 in the midgut of mosquitoes through injection of miR-1174 mimic and antagomir at adult stage [23]. Here, we synthesized miR-274 mimic and antagomir, but all injections of different dosages and time points could not effectively change the expression of miR-274 in the silk gland. Further, we tried the mimics and antagomirs of other miRNAs, but turned out to be failures without exception. Therefore, this simple and efficient technique, which does not require complex genetic manipulation, is not suitable for the functional study of miRNAs in this specialized organ (Fig. 4a and b).

The mature sequence of miR-30 accumulates in mammalian cells when *pol II* promoter was used to overexpress the precursor of miR-30 [25]. Here in this work, we realized the up-regulation of miR-274 in the MSG and PSG, through injection of transgenic overexpression plasmids overexpressing pre-miR-274, and it is beyond doubt that transgenic overexpression technology is an effective gain-of-function approach for miRNAs in silk gland (Fig. 4c). As competitive inhibitors, miRNA sponges are expressed under strong promoters and can strongly depress miRNA targets [16]. Combined with UAS/Gal4 system, artificial sponges have been widely used to down-regulate tissue−/stage-specific miRNAs in different species to create loss-of-function phenotypes (Fig. 4d). In this study, the whole-body *A4* promoter of silkworm was used to express the miR-274 sponge in silk gland, and only about 20% down-regulation was achieved in the silk gland (Fig. 1j), and the inhibition effect should be improved by using more efficient promoters or increasing the number of binding sites.

As the latest and most efficient genome editing technology, CRISPR/Cas9 system has been widely used in various organisms (Fig. 4e). Remarkable progress in genome editing of silkworm have been made by using CRIRSPR/Cas9 technology [26–29]. Our laboratory researchers have also implemented genome editing in the silkworm and even knocked out some important protein coding genes in the silk gland by CRISPR/Cas9 technology [24, 30]. However, up to now, no reports are available on the use of CRISPR/Cas9 system to study miRNAs in the silk gland of silkworm. We designed two gRNAs expressed simultaneously to guide the cleavage, and diverse deletions of bases were found at each gRNA binding site, which were similar to single gRNA-mediated knockout, but no fragment deletions were observed within the precursor of miR-274. The deletion of the bases exhibited a strong directivity, occurring upstream of the PAM structure of gRNA in the genomic sequence, which is similar to the knockout of *BmLMN* [24]. The CRISPR/cas9 targeting miR-274 vector is not injected for transient expression of the Cas9 protein in the silk gland. On the contrary, the strategy we used to knockout miRNA in silk glands can lead to the knockdown phenotypes for a long term because the positive strains of gRNAs and Cas9 protein can be steadily inherited, and the stable and simultaneous expression of gRNA and Cas9 is achieved by hybridization of the positive gRNA strain and the Cas9 strain, which stably expresses the Cas9 in the posterior silk gland during the whole larval stages of silkworm [24].

## Conclusion

Silk gland of *Bombyx Mori* is an ideal organ for exploring gene regulation and tissue remodeling. However, functional study of silk gland miRNAs depends on techniques compatible with this specialized organ. In this work, a practical technical system for studying the function of miRNAs in silk gland was established, which provides an important technical reference for the functional study of miRNAs and other noncoding RNAs in silk gland of *Bombyx mori*. The technical system and research strategy of this study will also be valuable for studying the functions of noncoding RNA in other insect organs and tissues.

## Methods

### RNA Extraction and Real-time Fluorescent Quantitative PCR Assay

The total RNA of tissues of silkworm (Dazao) was extracted by TRIzol (Ambion, U.S.A), and the concentration was determined by spectrophotometer (NanoDrop 2000). DNase I (Promega, U.S.A) was used to digest the genome DNA at 37 °C for 30 min. MiRNA reverse transcription kit (Clontech, U.S.A) was used to synthesize the cDNA, and the expression of miRNA-274 was detected by real-time fluorescent quantitative PCR (qPCR) on the instrument, ABI 7500 fast. The primers for qRT-PCR include qmiR-274-5p, TTTGTGACCGTCACTAACGGGCA; qmiR-274-3p, TCGTTTTGGCGATCGCAAAATG; qmiR-1175, AAGTGGAGTAGTGGTCTCATCG.

### Injection of MiRNA Mimics and Antagomirs

The mimics and antagomirs of miRNAs were synthesized in Dharmacon (U.S.A) and were dissolved with nuclease free water to 100 μM and 200 μM, respectively. To avoid repeatedly freezing and thawing, the dissolved reagents were packed into small tubes and stored at − 80 °C for use. Totally, each silkworm larva at D3 IL 5 was injected with 0.5 nmol miR-274 mimics or 1.0 nmol antagomir. The injection was carried out through the stomata on the surface of silkworm larvae. The non-injected larvae were set as the wild type control and those injected with NC mimic and NC antagomir served as negative control groups. All silkworms were fed with fresh mulberry leaves and the silk glands were collected about 48 h post injection for the extraction of total RNA and qPCR assay. The sequences of miRNA mimics and antagomirs injected here are: bmo-miR-274 mimic: UUUGUGACCGUCACUAACGGGCA; bmo-miR-274 antagomir, 5′ mU(*)mG(*)mCmCmCmGmUmUmAmGmUmGmAmCmGmGmUmCmA(*)mC(*)mA(*)mA(*)mA (3′-Chl); aae-miR-1175 mimic, AAGUGGAGUAGUGGUCUCAUCG.

### Design of MiR-274 Sponge and Construction of Transgenic Vector

Six sequences reverse complementary to miR-274 were linked by fragment GAUCG (underlined in the sponge below) to synthesize the miR-274 sponge, within which the bases 9–12 after the seed of each mature sequence were mutated to form a protuberance. The *pUC57* [*miR-274 sponge*] vector was synthesized at Beijing Genomics Institute (BGI) after adding the *BamH* I and *Not* I (TAKARA, Japan) sites at 5′ and 3′ end of the sponge sequence (highlighted in bold in the sponge sequence below), respectively. The recovered vector [*miR-274 sponge*] from digestion of *BamH* I and *Not* I was cloned into *pSL1180* [*Hr3/A4-Luc*] to obtain *pSL1180* [*Hr3/A4-miR-274 sponge*]. The recovered miR-274 expression cassette [*Hr3/A4-miR-274 sponge*] from *Asc* I (NEB, U.S.A) digestion was cloned into the *piggyBac* [*3 × P3*-*EGFP*] to construct the overexpression vector of miR-274 sponge, *piggyBac* [*3 × P3*-*EGFP*, *Hr3/A4-miR-274 sponge*] with the *3 × P3*-activated *green fluorescence* as a screening marker. When verified by *Asc* I digestion, the sponge plasmids were extracted with QIAprep Spin Miniprep Kit (QIAGEN, Germany) and measured by spectrophotometer (NanoDrop 2000). The packed plasmids with a concentration of about 400 ng/μL and a A260/A280 ratio about 1.8 were stored at − 80 °C for use. The synthesized miR-274 sponge: **CGGGATCC**ACUGCCCGUUGUGUGCCUCACAAACGAUCGACUGCCCGUUGUGUGCCUCACAAACGAUCGACUGCCCGUUGUGUGCCUCACAAACGAUCGACUGCCCGUUGUGUGCCUCACAAACGAUCGACUGCCCGUUGUGUGCCUCACAAACGAUCGACUGCCCGUUGUGUGCCUCACAAAC**GCGGCCGCAA.**

### Construction of transgenic miR-274 overexpression vector

The silkworm genome DNA served as the template to amplify the precursor sequence of miR-274 (miR-274-OE) with primers miR-274-OE-F: CGGGATCCTTTATCGTTTCCAGGGTGTCG (*BamH* I site underlined) and miR-274-OE-R: TTGCGGCCGCGCTCGCACCTTCCACCTTCT (*Not* I site underlined). The precursor fragment was cloned into pMD-19 T vector by TA cloning and verified by sequencing. After digestion with *BamH* I and *Not* I, the verified fragment was cloned into *pSL1180*[*Hr3/Ser1-Red*] and *pSL1180*[*Hr3/FibH-EGFP*], respectively, producing the recombinant plasmids *pSL1180*[*Hr3/Ser1-miR-274-OE*] and *pSL1180*[*Hr3/FibH-miR-274-OE*]. After digestion with *Asc* I, the expression cassettes [*Hr3/Ser1-miR-274-OE*] and [*Hr3/FibH-miR-274-OE*] were cloned into the transgenic vectors *piggyBac*[*3 × P3-Red*] and *piggyBac*[*3 × P3-EGFP*], respectively, generating the MSG-specific recombinant overexpression vector, *piggyBac*[*3* × *P3-Red,Hr3/Ser1-miR-274-OE*] and the PSG-specific overexpression recombinant vector, *piggyBac*[*3 × P3-EGFP, Hr3/FibH-miR-274-OE*]. After digestion by *Asc* I, both plasmids were extracted QIAprep Spin Miniprep Kit (QIAGEN, Germany) and measured by spectrophotometer (NanoDrop 2000) and the packed plasmids with a concentration of over 400 ng/μL L and a A260/A280 ratio about 1.8 were stored at − 80 °C for use.

### Injection of Transgenic Overexpression Vectors and Screening of Positive Individuals

Each transgenic overexpression plasmid above was mixed at a mole proportion of 1:1 with the plasmid *pHA3PIG*, an auxiliary vector of *piggyBac* transposase preserved in our laboratory. The mixture of plasmids was injected into D9L silkworm eggs with a microinjector within one hour after being laid and then the injection holes were sealed with non-toxic instant dry glue. The injected eggs were incubated at 25 °C and 90% relative humidity for hatching. After 9–10 days, fresh mulberry leaves were used to collect newly hatched silkworms. The larvae were cultivated to adults for intragroup mating to obtain eggs of the first filial generation (F1 generation). The eggs were incubated at 25 °C and 90% relative humidity for six days (the day when the green spot emerges), when the transgenic positive individuals were screened by using macro-electro-fluorescence microscopy MVX10 (Olympus, Tokyo, Japan). The EGFP marker was screened under blue light produced by excitation filter BP460-480HQ and barrier filter BA495-540HQ. The Red gene marker was screened under green light produced by excitation filter BP535-555HQ and barrier filter BA570-625HQ.

### Design of GRNA and Construction of Transgenic GRNA Expression Vector

The sequence containing pre-miR-274 was input into the CCtop website (https://crispr.cos.uni-heidelberg.de/) [31] to determine the binding sites of gRNA with the structure G(N20)GG. The gRNAs with low off-target rate were screened at both ends of the precursor, and were named gRNA1 and gRNA2. After adding the terminal bases (in bold), the primers of gRNA1 and gRNA2 were synthesized in BGI. gRNA1-spacer-F: **TGCA**GCAGTAAGTGCGACGTGAGC, gRNA1-spacer-R: **AAAC**GCTCACGTCGCACTTACTGC, gRNA2-spacer-F: **TCCG**GCGGCTCACGAGCGGGGGAG and gRNA2-spacer-R: **AAAC**CTCCCCCGCTCGTGAGCCGC.

The gRNA spacer sequences were annealed to form double-stranded DNA. After digestion by *Aar* I (Thermo fisher, U.S.A), the gRNA1-spacer was ligated to *pUC57* [*U6-2gRNA*] vector to generate the intermediate vector *pUC57* [*U6-miR-274-gRNA1*], which was further verified by sequencing. After digestion by *Bbs* I (NEB, U.S.A), the gRNA2-spacer was ligated to the vector *pUC57* [*U6-miR-274-gRNA1*] to form the vector, *pUC57*[*U6-miR-274-2gRNA*], which was then digested with *Asc* I (NEB, U.S.A) to recover the 2gRNA expression cassette, namely [*U6-miR-274-2gRNA*]. The purified expression cassette [*U6-miR-274-2gRNA*] was finally ligated to the transgenic vector *piggyBac*[*3 × P3-EGFP*], generating the transgenic 2gRNA overexpression vector *piggyBac*[*3 × P3-EGFP, U6-miR-274-2gRNA*].

### Screening and Verification of MiR-274 Knockout Strains

The positive transgenic individuals of miR-274-2gRNA were cultivated to adults, which were then crossed with the PSG-specific Cas9 transgenic expression strain. Positive F1 individuals were screened under the macro-electro-fluorescence microscopy at day 6 embryo according to the light in the eyes: no light in wild type (WT), red light in the Cas9 expression line (PSG-Cas9), green light in the double gRNA expression line (miR-274-2gRNA), and both red and green light in the knockout line (△miR-274-PSG). The knockout line △miR-274-PSG and the controls were cultivated to D3 IL5, when the PSG was dissected out for the extraction of genomic DNA using the tissue DNA extraction kit (Omega, U.S.A). The sequence covering the knockout site of miR-274 was amplified with primers, pF: CCAGTAGCGTCCATTTCTTCC and pR: CATACTGTGAACTGGTGTCCCTA. All PCR products were submitted to TA cloning and sequencing, followed by analysis with software BioEdit.

## Data Availability

The datasets used and/or analyzed during the current study are included in this published article and are available from the corresponding author on reasonable request.

## Abbreviations

ASG: Anterior silk gland
CRISPR: Clustered regularly interspersed palindromic repeats
CRISPR)/Cas9: CRISPR-associated protein 9
D3 IL5: Day 3 of the fifth instar
gRNA: Guide RNA
MSG: Middle silk gland
NC: Antagomir, negative control antagomir
NC: Negative control mimics
OE: Overexpression
PAM: Protospacer adjacent motif
PCR: Polymerase chain reaction
PSG: Posterior silk gland
qPCR: Real-time fluorescent quantitative polymerase chain reaction
WT: Wild type
